# Bibliography

**Published:** 1891-01

**Authors:** 


### BIBLIOGRAPHY.

THE MICRO-ORGANISMS OF THE HUMAN MOUTH; the Local and General Diseases which are caused by them. By WILLOUGHBY D. MILLER, D.D.S., M.D. 128 illustrations, one chromo-lithographic and two photo-micrographic plates. The S. S. White Dental Manufacturing Company. Philadelphia, 1890.

English readers have waited with a good deal of interest the authorized edition of this work, directly from the hands of the author. Its preparation must have involved Professor Miller in a labor that exceeded that of the original German edition, as a translation is more laborious than the original work; but it has been most successfully accomplished, and it can now be judged with intelligence and discrimination.

The preface opens with an allusion to a great and much-neglected truth which every one, without exception, should heed, that the mouth is a culture place unsurpassed, and should be the principal battle-ground in the warfare against disease.

"It has been established beyond all question that myriads of micro-organisms are constantly present in the human mouth, and that these, under favorable circumstances, are capable of manifesting an action of the utmost significance upon the local as well as the general health of the patient. Not alone are they responsible for the vast majority of those diseases of the teeth and contiguous parts which the dental profession is called upon to treat, but they also give rise to other local and general disorders of the most serious nature. . . .

"The existence of a most excellent nursery for bacteria at the very portal of the human body is a fact which has only recently begun to receive the attention which its importance demands."

The first three chapters are devoted to the instruction of those who have not given special attention to bacteriological studies. This is very essential, for otherwise the contents would be incomprehensible to many. Even with these very lucid pages, it may be presumed that one not familiar with micro-organisms will still find it difficult to arrive at a realizing sense of these invisible forms.

A consoling fact is alluded to on page 13, which deprives these low forms of life of continuing power. The author alludes to the struggle for supremacy as follows: "The principle of the struggle for existence and of natural selection plays an important, though not fully understood, part in the life of bacteria. If we bring a number of different kinds of bacteria into a common culture medium, it will be found that they do not all develop with equal rapidity; one kind will always attain the supremacy. If we infect a second nutrient medium with this culture and continue the operation through a series of generations, we may find that at last only one kind remains, the others having been crowded out. It depends upon the composition of the solution, the temperature, and various other conditions, as to which species will gain the victory. It may also happen that at first one kind prevails, and that later, through a change in the character of the medium, another kind gains the upper hand; or, again, in one part of the medium—for example, on the surface—one kind may prevail, in another part another. In the human mouth this struggle for existence seems to play an important part." . . . Again: "The growth and ferment activity of bacteria are always more or less influenced by their own waste products. Lactic acid fermentation not only ceases when the acidity of the solution has reached 0.75 to 0.80 per cent., but the bacteria themselves are often destroyed by the action of the acid which they have produced."

The following quotation will explain many peculiar and diverse influences so frequently found puzzling pathologists. "Pathogenic bacteria exert very different and diverse influences upon different species of animals. An animal of one species may manifest symptoms of disorder soon after inoculation, while members of another species may remain altogether unaffected by it. Even different varieties of one and the same species do not always manifest the same degree of susceptibility when inoculated with the same bacterium; *e.g.*, house mice inoculated with the bacillus of mouse septicæmia in a skin pocket, succumb within forty to sixty hours, whereas field mice suffer no inconvenience from the inoculation. Koch explains this phenomenon by the difference of the blood of these two nearly-related varieties."

Under the head of "Putrefaction" the following, of interest, is selected: "The appearance of putrefactive products in any fermentation does not depend alone upon the kind of fermentation and the kind of bacterium, but more especially upon simultaneously-occurring processes which have little or nothing whatever to do with the decomposition of the decaying substance. If, for instance, a tooth-pulp decomposes in a closed root-canal, an incredible amount of bad-smelling gas may be formed, so that, on opening the pulp-chamber, the whole room will be impregnated with the odor. But if a tooth-pulp be allowed to decompose in the open air, hardly any disagreeable smell will be noticed, although the decomposition of the nutrient medium caused by the fungi may be identical in both cases.

Under "Nutrient Media for Bacteria in the Oral Cavity," the author considers the liability of pure saliva undergoing putrefaction, and says, "Pure saliva exposed to the air undergoes putrefaction, not as rapidly, however, as is generally asserted in text-books of physiology; on the contrary, sometimes very slowly, so that no bad smell may be detected before several days have elapsed. Only when the saliva is mixed with organic matter from the mouth does it show marked signs of putrefaction. . . . The view entertained by many dentists that saliva possesses antiseptic properties seems to me to be unfounded."

Those who carelessly bruise the gums with clamps, ligatures, or bands may find the following more than suggestive; it may be a warning that should not go unheeded. "The gums, when irritated by accumulations of tartar or food, or by sharp edges, protruding fillings, roots, etc., furnish particularly favorable conditions of nutrition for certain species of bacteria. The apparently strictly obligatory parasitic bacteria of the mouth, *Spirochæte dentium* and *Spirillum sputigenum*, find here the peculiar conditions essential to their development."

The methods of bacterial investigation, covering many pages, is of deep and vital interest to the worker in this direction, but must be passed over with the simple remark, that the clearness of illustration should make it a simple matter for one familiar with the microscope to follow the methods described.

On the biological studies of the "Bacteria of the Human Mouth," the author calls special attention to the well-known form, *Lepthotrix buccalis*, and disposes of the claims of certain histologists in this summary manner. "*Lepthotrix buccalis* is a name chosen by Robin for those organisms in the human mouth which were formerly described as animalculæ, tooth animalcules, Bühlmann's fibres, denticolæ, etc. . . . Morphologically as well as physiologically considered, *Lepthotrix buccalis* has been regarded as a veritable wonder. It has been said to perforate and split up teeth, its elements to cause all kinds of diseases in the oral cavity, to penetrate into the lungs, the stomach, and other parts of the body, and everywhere to manifest a destructive influence. As absolutely nothing was known concerning the biology and pathogenesis of this organism, all sorts of wonderful properties were ascribed to it. It is high time to banish this confusing name from bacteriological writings."

The whole of this portion of the work should be closely followed. It is profusely illustrated, and will give a clearer idea of the various forms and characteristics than would be possible by any number of quotations. It is impossible, however, to omit a portion of his views on "Chromogenic Mouth Bacteria." "I have found in the human mouth and isolated no less than eight different kinds of bacteria, which produce a yellow pigment, not including the well-known yellow sarcina. These bacteria are themselves yellow, but do not impart any color to the culture medium. . . .

"I have made a great many attempts to cultivate the supposed bacterium of green stain, but so far without success. I have indeed isolated five different species of bacteria from the mouth which impart a green color to the culture-media, although themselves colorless, but I do not bring any of them into causal connection with the green stain, since, as far as my observation goes, the bacterium of green stain, if there be such a thing, does not grow on gelatine. . . .

"I have recently isolated a bacterium from the mouth . . . which, cultivated on the surface of nutritive agar-agar, imparts to the medium . . . a yellowish-brown color which gradually darkens."

After describing other forms and some experiments that the "black color of the putrid pulps might be accounted for by the formation of the sulphide of iron," he says, "Whether sulphide of iron may be formed through decay of the dentine or enamel in sufficient quantity to aid in discoloring the same, I cannot say; at present I doubt it."

On page 96 the important topic is discussed, "The Bacteria of Diseased Pulps." After stating that he can "only communicate the results" of investigations, he says, "It has, moreover, appeared to me that in cultures from such pulps I have not, as a rule, found the large number of different kinds of bacteria which may usually be found in the mouth. . . . It may, therefore, readily occur that a putrid pulp does not contain a single bacterium capable of development."

The following from the same chapter is so at variance with generally-received opinions that some may find it difficult to accept it: "It often happens that a tooth, which has occasioned no disturbance for years, in spite of a necrotic pulp, will exhibit a severe inflammation of the pericementum a few hours after the pulp chamber has been opened. . . .

"An attempt has been made to ascribe this very unpleasant result to an infection of the pulp occasioned by germs from the air. . . . This idea deserves to be ranked among the many other wonderful theories of olden times." The question is then argued on the basis that the "pure air in Berlin was found to contain, on the average, 0.1 to 0.5 bacteria per litre," and then concludes that "pericementitis, after opening into a pulp-chamber, is due solely to the carelessness or helplessness of the operator. He either forces particles of the putrid pulp through the foramen apicale, or introduces new infected fermentable matter into the root-canal from without, or occasions an infection by means of unclean instruments."

While experiments have long since demonstrated that air in certain localities is nearly devoid of bacteria, and in others they appear to exist "in minute clouds, with interspaces devoid of any," it is not safe to assume that the ingress of air plays no part in the infection that does certainly take place. The writer of this is not prepared to accept the conclusion that this is due to carelessness. Those who are familiar with the treatment of this class of teeth for the past forty years are perfectly cognizant of the fact that no class of operations have received more attention or were more dreaded than these. The certainty that the opening into a so-called dead tooth would eventuate in pericementitis in a few hours, caused the dentist to hesitate, and when forced to act to use the utmost caution. Before the days of known antisepsis and more intelligent ideas in regard to microbic infection, it was natural to ascribe this sudden change to spores in the air. Admitting the calculations of the author to be correct, the explanation fails to meet all the difficulties in the case. Careful operators were as cautious in the "olden times" as now. The opening was made, and the subsequent treatment was performed by slow and oftentimes tedious repetitions of engagements, and yet, despite this care, pericementitis followed in most cases. The use of creosote and carbolic acid was almost universal, and this, according to our author, will prevent bacteria in one to five thousand. What is the result now? In the same hands, followed immediately with careful antiseptic treatment, the percentage of loss is reduced to almost nothing; indeed, the possibility of pericementitis is not ordinarily taken into consideration. Is this due to more careful sterilization of instruments, or to exclusion of air? It will not do to assert too much, and it would be equally rash to accept the author's conclusions without further evidence.

In chapter vi., page 119, the author opens the subject of "The Decay of the Teeth." As this is the basis of the entire work, it is, naturally, the most interesting to the dental reader. There is a very liberal elucidation of the older theories of caries before giving the "Original Investigations of the Decay of the Teeth."

It is difficult to pass over this, the most important in the entire book; but to quote sparingly would be to give imperfect renderings of this work, where every page is filled with matter of vital moment, and to quote in full would be impossible in the limits allowed for this review. The subject must be left to the careful reader, with the assurance that no one unprejudiced can arise from the examination of the text, illustrated as it is, without a conviction that this labor has been most thoroughly done, and that all causes of error have been eliminated from the conclusions as far as human foresight and care could accomplish it. Miller's work in this particular direction has been so long before the world, that the idea may prevail that this book is simply a repetition of his periodical work. The fact is, that the old results have been so enriched by constant reinvestigations, that it seems to have all the character of an orignal production. This is increased by the addition of many illustrations, and especially of four photo-micrographic pictures,—

*a.* "A longitudinal section of decayed dentine, showing infection with micrococci, bacilli, and threads."

*b.* "Longitudinal section of decayed dentine, showing infection with micrococci."

*c.* "Diagonal section of decayed dentine. Infection with micrococci, bacilli, and screw forms."

*d.* "Longitudinal section of dentine artificially decayed. Infection with bacilli."

These are very perfect, and illustrate most satisfactorily the several conditions. The credit of this most excellent piece of work in micro-photography belongs to Dr. Carl Günther, of Berlin.

The scientific world is familiar now with the fact that Dr. Miller, through long, patient, and ever repeated investigations, demonstrated the cause of caries and, as it were, proved his own conclusions by producing caries out of the mouth, and this to the satisfaction of all capable of judging. Nothing was left unattempted that seemed to militate against observed facts. The tests were conclusive, and in these pages the story is told, and is from beginning to end one of the most remarkable exhibits of patient research, perseverance amid many difficulties, combined with a determined purpose to leave nothing unattained.

The importance which the author gives to the oral cavity as a nursery for bacteria, already quoted, leads to the impression that he will devote considerable attention to prophylactic measures, and this is borne out in the very thorough treatment of this important subject, "Prophylaxis of Dental Decay." In this the relative value of different agents is considered in detail, followed by the antiseptic action of filling-materials, a subject made familiar by recent publications of the author.

Under sterilization of teeth for implantation the following is taken as expressing Dr. Miller's views. After quoting cases showing the danger of this operation, he says, "In my judgment the dentist who undertakes the above operation without having previously convinced himself that the tooth to be implanted is in a perfectly aseptic condition, or most certainly contains no specific germs, commits a serious wrong.

"But how can we thoroughly sterilize a tooth? We must, in the first place, reconcile ourselves to the fact that a complete sterilization of a suspected tooth with the conservation of the periosteum is absolutely out of the question. It is not alone necessary to sterilize the surface of the tooth, but the whole substance of the tooth; and since under certain circumstances the cement-lacunæ, as well as the dentinal tubules, may contain bacteria, it is not to be supposed for a moment that we can reach and kill them in these portions without injuring the pericementum. The transplantation or implantation of a tooth with living pericementum is, therefore, practicable only in such cases where the tooth is taken directly from a living, perfectly healthy individual, and placed at once, under antiseptic precautions, into the alveolus. . . . The usual method of disinfection, placing the teeth in dilute solutions of bichloride of mercury for a few minutes or even hours, gives no guarantee that the teeth are made absolutely sterile. . . . A certain sterilization, however, can be effected by heat alone, either by boiling water or by steam at 100° C. . . . It will be necessary to repeat the sterilization process two or three times at intervals of about twelve hours."

It would give great pleasure to the writer and would add interest to this article to quote from Part II., "The Pathogenic Mouth Bacteria and the Diseases which they Produce," but space is not at command for further comment. It is thorough and most valuable, and should be carefully studied. Indeed, this can be said of the entire book.

In concluding this review the feeling is prominent that the closing chapters open up to all classes of general and special workers in diseased conditions a field of thought that cannot with safety be neglected. Every line of it carries its own lesson, a lesson filled with important bearings on pathological studies and calculated to arouse serious conceptions of duty. The dental profession has received many such from inside as well as outside the ranks. The recent article in the August and September numbers of this journal, by L. Duncan Bulkley, M.D., is an important contribution to this subject.

This work as a whole cannot be read in a day, a week, or a month; but must be studied in every part with a definite purpose. That this study will be facilitated by some previous knowledge of the subject is true, but the clear descriptive text and still clearer illustrations make it a comparatively easy task for the untrained in microscopic work to follow the author.

Any review must necessarily be incomplete; but sufficient has been given to show that in its line of work it has no equal; indeed, nothing comparable to it has appeared. That it covers the whole subject could not be expected, as bacteriology is comparatively in its infancy, and Professor Miller himself shows that he considers his labors but at the beginning, for he says, "Any one, therefore, who continues the search for oral bacteria by means of the modern culture method, for a long period of time, will continually meet with new kinds."

It is impossible to leave the book without expressing the satisfaction with the results obtained by the publishers, S. S. White Dental Manufacturing Company.

The additional micro-photographs, not in the German edition, add greatly to the elucidation of dental caries. These, together with the unexceptionable character of the production in the line of book-making, gives a work above criticism in every respect.

